# Abnormal transaminase and lipid profiles in coexisting diseases in patients with fatty liver: a population study in Sichuan

**DOI:** 10.1042/BSR20211769

**Published:** 2021-12-17

**Authors:** Wei Jiang, Chang-hai Liu, Dongbo Wu, You-Juan Wang, Hong Tang

**Affiliations:** 1 Center of Infectious Diseases, West China Hospital, Sichuan University, Chengdu, China; 2 Division of Infectious Diseases, State Key Laboratory of Biotherapy and Center of Infectious Disease, West China Hospital, Sichuan University, Chengdu, China; 3 Health Management Center, West China Hospital of Sichuan University, Chengdu, China

**Keywords:** coexisting diseases, fatty liver, lipid metabolism, multivariable logistic regression

## Abstract

Among chronic liver diseases, fatty liver has the highest incidence worldwide. Coexistence of fatty liver and other chronic diseases, such as diabetes, hepatitis B virus (HBV) and *Helicobacter pylori* (Hp) infection, is common in clinical practice. The present study was conducted to analyze the prevalence and association of coexisting diseases in patients with fatty liver and to investigate how coexisting diseases contribute to abnormal transaminase and lipid profiles. We enrolled participants who were diagnosed with fatty liver via ultrasound in the physical examination center of West China Hospital. Multivariable logistic regression was used to determine the adjusted odds ratios (ORs). We found that 23.6% of patients who underwent physical examinations were diagnosed with fatty liver. These patients had higher risks of metabolic syndrome (MetS), type 2 diabetes mellitus (T2DM), and hypertension and a lower risk of HBV infection. The risks of Hp infection and hyperthyroidism did not statistically differ. When fatty liver coexisted with T2DM, MetS and thyroid dysfunction, it conferred a higher risk of elevated transaminase. Fatty liver was positively correlated with triglycerides, cholesterol and low-density lipoprotein cholesterol (LDL-C) and negatively correlated with HBV; thus, HBV had a neutralizing effect on lipid metabolism when coexisting with fatty liver. In conclusion, patients with fatty liver that coexists with T2DM, MetS and thyroid dysfunction are more prone to elevated transaminase levels. Patients with both fatty liver and HBV may experience a neutralizing effect on their lipid metabolism. Thus, lipid alterations should be monitored in these patients during antiviral treatment for HBV.

## Introduction

Fatty liver is a disease involving hepatocellular degeneration due to excessive deposition of liver fat, with the global incidence being approximately 24%, making it the most prevalent chronic liver disease worldwide [1–4]. Compared with the general population, patients with fatty liver have higher liver-related morbidity and mortality and tend to exhibit extrahepatic diseases such as *Helicobacter pylori* (Hp) infection, type 2 diabetes mellitus (T2DM), metabolic syndrome (MetS), insulin resistance (IR) and thyroid dysfunction, thus creating a considerable health and economic burden worldwide and often resulting in a poor quality of life for these patients [5]. Additionally, patients with fatty liver often experience symptoms of MetS, T2DM and obesity [6,7]. The European Association for the Study of the Liver has proposed a new definition of ‘metabolic fatty liver disease’ (MAFLD), which unifies these conditions [8]. However, the differences between pure fatty liver and diseases that coexist with fatty liver, such as the degree of loss of liver function and the degree of dyslipidemia, require further research. For example, clinicians should consider whether a concomitant disease exists when addressing abnormal liver functions or lipid profiles in patients with fatty liver and *vice versa*.

Hp is a cofactor in developing peptic ulcers, gastric cancer and gastric mucosa-associated lymphoid-tissue lymphoma and is one of the most frequent gastrointestinal infections in humans [9]. Several recent observational studies have examined the association between Hp infection and risk of nonalcoholic fatty liver disease (NAFLD) [10–14]. A systematic review and meta-analysis of observational studies suggested that Hp infection was associated with a mildly increased risk of both prevalent and incident NAFLD in middle-aged individuals [15]. However, the random-effects odds ratio (OR) and 95% confidence interval (CI) in the report were near 1 (OR: 1.20; 95% CI: 1.07–1.35), and most of the results of the included studies failed to establish a causal association between Hp infection and NAFLD [15]. Thus, further studies are needed to further validate the relationship between these two diseases.

Thyroid hormones are reported to have prominent effects on hepatic fatty acids, cholesterol synthesis and metabolism. Many researchers suggest that thyroid hormones may be closely related to the fatty liver pathogenesis [16–18]. However, other researchers believe that it remains unclear whether thyroid dysfunction is associated with fatty liver [19,20]. Additionally, debates are ongoing in the hepatology field regarding the use of thyroid hormones as anti-steatohepatitis (NASH) and anti-fibrosis drugs [21–23]. Therefore, more evidence is needed to support the relationship between thyroid dysfunction and fatty liver.

Chronic hepatitis B (CHB) is another significant cause of chronic liver disease worldwide, and is caused by hepatitis B virus (HBV) infection [24]. Because of the high prevalence and increased diagnosis, coexistence of fatty liver with HBV is frequently observed in clinical practice. A previous study assessed the clinical outcomes and prognostic risk factors for CHB and NAFLD and found that patients with concomitant NAFLD and CHB developed liver-related outcomes or death more quickly than did patients with CHB alone [25]. The prognostic risk of both NAFLD and CHB is well established for patients with chronic liver disease, and patients with both CHB and fatty liver disease must be appropriately managed [26]. However, clinical study data are inconclusive regarding whether fatty liver interferes with HBV infection or whether HBV infection affects fatty liver formation in patients with CHB. Elevated transaminase usually leads to hepatocytic damage and thus fibrosis and hepatocellular carcinoma. In patients with multiple coexisting liver diseases, the most common causes of aminotransferase elevations are immune-active hepatitis B (48.4%), alcohol consumption (30.8%) and NAFLD (24.7%). However, among patients with HBV DNA levels that are persistently <2000 IU/ml, the most common causes are NAFLD and alcohol consumption [27]. Therefore, careful assessment is needed to identify other potentially modifiable conditions before starting antiviral treatment for HBV. However, elevated transaminase levels due to coexisting diseases in patients with fatty liver are rarely reported. Moreover, changes in lipid metabolism require additional research in patients with fatty liver combined with other diseases, especially hepatitis B.

In the present study, we first analyzed the prevalence and association of coexisting diseases in patients with fatty liver disease. Second, we investigated how the coexisting disease contributed to abnormal transaminase and lipid profiles.

## Methods

### Study design and population

Patients were selected from the digital records of the health examination center of West China Hospital of Sichuan University between 1 January 2014 and 31 December 2017. The inclusion criteria were patients who underwent an abdominal ultrasound. The exclusion criteria were patients with hepatitis C. Patients’ information, including sex, age, residence, liver biochemistry results, blood glucose, blood pressure, blood lipids and other blood tests, were automatically extracted from the hospital information system. Patient records/information were anonymized prior to analysis. Anthropometric measurements included weight, height and waist circumference. Body mass index (BMI) was computed as weight divided by height in kg/m^2^. Being overweight was defined as a BMI > 23 kg/m^2^ [28]. The Institutional Review Board Committee of West China Hospital of Sichuan University approved the study protocol. The study was performed in accordance with the ethical guidelines in the Declaration of Helsinki and the International Conference on Harmonization Guidelines for Good Clinical Practice. All participants provided informed consent.

### Diagnosis

Fatty liver was diagnosed based on abdominal ultrasonography [29]. HBV infection was defined as serum hepatitis B s antigen (HBsAg) positive [30]. MetS was characterized by an elevated waist circumference, elevated triglycerides (≥150 mg/dl), reduced high-density lipoprotein (HDL)-C (<40 mg/dl in men; <50 mg/dl in women), elevated blood pressure (systolic ≥ 130 and/or diastolic ≥ 85 mmHg), and elevated fasting glucose (≥100 mg/dl) [31]. Diabetes was diagnosed by measuring patients’ hyperglycemia level with no acute physiological stress or existing symptoms of hyperglycemia (fasting plasma glucose ≥ 7.0 mmol/l) [32]. Hp infection was diagnosed based on a positive C^14^ urea breath test (disintegrations per minute [DPMs] > 100) [33]. Subclinical hyperthyroidism was diagnosed when the serum thyroid stimulating hormone (TSH) was <4.2 mU/l, and the free T4 and T3 concentrations were normal. Overt hyperthyroidism was diagnosed when patients had low serum TSH and normal free T4 and total T3, but increased free T3 (7.5 pmol/l) [34].

### Statistical analysis

Continuous data are expressed as means ± standard deviation; categorical variables are expressed as percentages. In the univariate comparisons, we used Student’s *t* test and analysis of variance with Bonferroni adjustments for continuous samples and a chi-square test or Fisher’s exact test for the qualitative samples. Risk factors for fatty liver were analyzed via logistic regression analysis; covariates with *P*<0.05 in the univariate analysis were further analyzed (entered model). All possible confounding factors were assessed considering biological plausibility by three models to adjust for these confounding risk factors. Model 1 adjusted for age. Model 2 adjusted for age and BMI. Model 3 adjusted for age, BMI, fatty liver, Hp infection, thyroid function, osteoporosis, hypertension, T2DM and MetS. Complete case analyses were used for missing data because the statistical packages excluded individuals with missing values. Statistical analyses were performed using SPSS software, version 20.0 (SPSS, Chicago, IL, U.S.A.). A two-sided *P*<0.05 was considered statistically significant.

## Results

### Participants’ selection and characteristics

**Figure 1:**
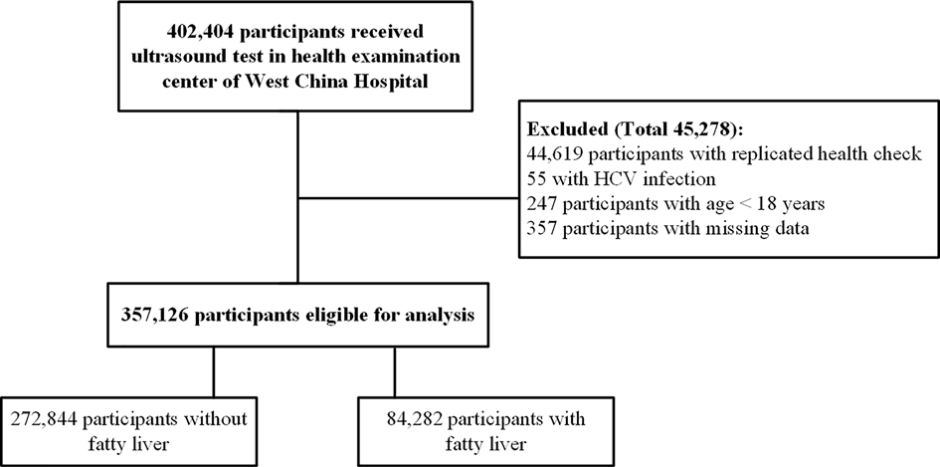
Participants’ selection and number of health check for included participants

We included 402404 patients who underwent liver ultrasonography in the physical examination center of West China Hospital from 2014 to 2017. After excluding 44619 patients with replicated health checks, 55 with hepatitis C virus infections, 247 aged <18 years, and 357 with missing data, 357126 patients were eligible for the final analysis (Figure 1).

The patients’ average age was 43.81 ± 12.45 years. The prevalence of fatty liver was 23.60% as per ultrasound diagnosis. Patients with fatty liver were more likely to be men (80.90 vs. 50.00%, *P*<0.001) and urban residents (52.43 vs. 50.60%, *P*<0.001) and were characterized by a higher BMI, total cholesterol, creatinine, bilirubin, aspartic transaminase (AST), alanine transaminase (ALT), γ-glutamyl transferase (GGT) and low-density lipoprotein (LDL) cholesterol compared with those without fatty liver (Table 1). Patients with fatty liver were more likely to have MetS (36.80 vs. 5.90%, *P*<0.001), Hp infection (34.01 vs. 32.92%, *P*=0.001) and diabetes (10.84 vs. 2.30%, *P*<0.001) than were those without fatty liver (Table 1).

**Table 1:** Estimated* mean values (95% CI) and adjusted* proportion (95% CI) of baseline characteristics of study participants

	Total	Fatty liver (−)	Fatty liver (+)	*P*-value
**Demographics**				
Number, *n* (%)	357126 (100.00%)	272844 (76.40%)	84282 (23.60%)	
Age (years)	43.81 ± 12.45	43.13 ± 12.65	46.00 ± 11.47	0.747
Male gender (%)	57.30%	50.00%	80.90%	<0.001
Urban residents (%)	51.01%	50.60%	52.43%	<0.001
BMI (kg/m^2^)	23.53 ± 1.40	23.32 ± 1.43	24.23 ± 1.01	<0.001
Normal (%)	63.73%	73.90%	30.55%	
Overweight (%)	28.40%	19.00%	58.70%	
Obesity (%)	3.12%	0.80%	10.70%	
**Biological indicators**				
Triglycerides (mg/dl)	1.57 ± 0.36	1.75 ± 0.26	1.52 ± 0.37	<0.001
Total cholesterol (mg/dl)	4.86 ± 0.22	4.84 ± 0.23	4.92 ± 0.19	<0.001
Creatinine (mg/dl)	70.71 ± 10.89	69.06 ± 11.05	76.07 ± 8.33	<0.001
Bilirubin (mmol/l)	14.53 ± 1.20	14.35 ± 1.21	15.10 ± 0.95	<0.001
AST (IU/l)	25.34 ± 2.65	24.94 ± 2.70	26.66 ± 1.96	<0.001
ALT (IU/l)	27.58 ± 7.14	26.61 ± 7.18	30.74 ± 6.02	<0.001
GGT (IU/l)	32.91 ± 11.68	31.15 ± 11.85	38.64 ± 8.99	<0.001
HDL cholesterol (mg/dl)	1.45 ± 0.18	1.48 ± 0.18	1.37 ± 0.14	<0.001
LDL cholesterol (mg/dl)	2.77 ± 0.18	2.75 ± 0.19	2.84 ± 0.15	<0.001
Platelets (*10^9^/l)	194.21 ± 13.96	195.96 ± 14.36	188.53 ± 10.79	<0.001
LSM (kPa)	4.68 ± 0.26	4.64 ± 0.27	4.79 ± 0.19	<0.001
CAP (dB/m)	242.55 ± 16.26	239.75 ± 16.75	249.95 ± 12.10	
**Comorbidities (%)**				
MetS	13.10%	5.90%	36.80%	<0.001
Diabetes mellitus	4.30%	2.30%	10.84%	<0.001
Hypertension	12.95%	9.8%	22.97%	0.001
HBsAg-positive	8.31%	8.61%	7.41%	<0.001
Thyroid disfunction	17.41%	17.42%	16.95%	0.001
Hp infection	33.18%	32.92%	34.01%	0.001

Abbreviations: CAP, controlled attenuation parameter; LSM, liver hardness measurement.

* Adjusted for age and sex. Values are expressed as mean ± standard deviation or percentages.

### Fatty liver was associated with a lower risk of HBV and a higher risk of MetS, T2DM and hypertension

Patients with fatty liver presented a lower prevalence of HBV than did patients without fatty liver (7.41 vs. 8.61%, *P*<0.001; Table 1). The adjusted OR (aOR) for HBV infection was 0.75-times lower (95% CI: 0.70–0.80, *P*<0.001, model 1) for individuals with fatty liver than for those without fatty liver. After adjusting for BMI, Hp infection, thyroid function, hypertension, T2DM and MetS, the odds of HBV infection in individuals with fatty liver were reduced to 0.71 (95% CI: 0.65–0.76, *P*<0.001, model 3; Table 2). The odds of T2DM, hypertension and MetS were higher for individuals with fatty liver than for those without fatty liver in model 3 (aOR: 2.73, 95% CI: 2.48–3.00, *P*<0.001; aOR: 1.61, 95% CI: 1.52–1.70, *P*<0.001; aOR: 3.74, 95% CI: 3.52–3.96, *P*<0.001, respectively; Table 2). The odds of Hp infection and hyperthyroidism were higher in patients with fatty liver than in patients without fatty liver (model 1); however, this significance was counteracted after adjusting for various factors in model 3 (Table 2).

**Table 2:** aORs by fatty liver status for HBsAg, Hp infection, thyroid function, T2DM, hypertension and MetS

	Fatty liver (−)^*^	Fatty liver (+)		Fatty liver (+)		Fatty liver (+)	
		Model 1	*P*-value	Model 2	*P*-value	Model 3	*P*-value
**HBV** (+)	Reference	0.75 (0.70–0.80)	<0.001	0.68 (0.63–0.73)	<0.001	0.71 (0.65–0.76)	<0.001
**Hp infection** (+)	Reference	1.05 (1.01–1.09)	<0.001	0.99 (0.95–1.04)	0.731	0.99 (0.95–1.03)	0.681
**Subclinical hyperthyroid** (+)	Reference	1.13 (1.10–1.15)	<0.001	1.03 (1.00–1.06)	0.033	1.04 (0.97–1.11)	0.244
**Overt hyperthyroid** (+)	Reference	1.53 (1.30–1.81)	<0.001	1.21 (1.01–1.46)	0.043	1.25 (0.84–1.86)	0.279
**T2DM** (+)	Reference	4.59 (4.24–4.97)	<0.001	4.04 (3.69–4.41)	<0.001	2.73 (2.48–3.00)	<0.001
**Hypertension** (+)	Reference	2.24 (2.13–2.36)	<0.001	1.44 (1.36–1.52)	<0.001	1.61 (1.52–1.70)	<0.001
**MetS** (+)	Reference	8.21 (7.81–8.62)	<0.001	3.92 (3.71–4.13)	<0.001	3.74 (3.52–3.96)	<0.001

Model 1: stratified by sex, resident, gender, year of health check and adjusted for age.

Model 2: model 1 further adjusted for BMI.

Model 3: model 2 further adjusted for Hp infection, thyroid function, osteoporosis, hypertension, T2DM and MetS.

* Patients without fatty liver as reference.

### Effects of fatty liver and various concomitant diseases on liver function

**Figure 2:**
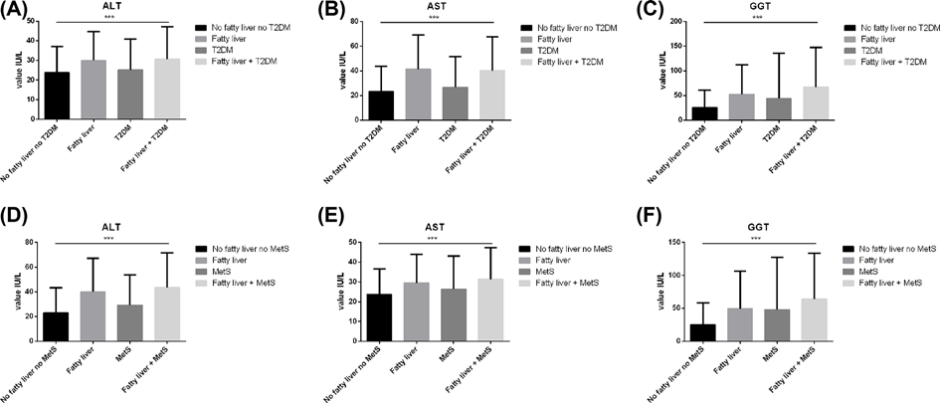
Analysis of ALT, AST and GGT levels in participants with fatty liver, T2DM and MetS (**A–C**) Mean value of ALT, AST and GGT values were lowest in patients without fatty liver or T2DM (*n*=266813), and highest in patients with both fatty liver and T2DM (*n*=9091). In addition, they were higher in patients with fatty liver without T2DM (*n*=75067) than in patients with T2DM without fatty liver (*n*=6155). (**D–F**) Mean value of ALT, AST and GGT values were lowest in patients without fatty liver or MetS (*n*=257875), and were highest in patients with both fatty liver and MetS (*n*=29094). In addition, they were higher in fatty liver without MetS (*n*=55064) than in patients with MetS without fatty liver (*n*=15093). *** means *P*<0.001.

The aORs were calculated by multivariant analysis in association of elevated ALT, AST and GGT levels in patients with fatty liver with or without coexisting diseases, including HBV, T2DM, MetS, Hp infection, hypertension and thyroid dysfunction. Using patients without fatty liver or T2DM as references, patients with both fatty liver and T2DM had significantly higher risks of elevated AST and GGT than did those with fatty liver but without T2DM (AST: aOR: 3.10, 95% CI: 2.90–3.32, *P*<0.001 vs. aOR: 2.37, 95% CI: 2.28–2.46, *P*<0.001; GGT: aOR: 3.74, 95% CI: 3.56–3.92, *P*<0.001 vs. aOR: 2.50, 95% CI: 2.44–2.56, *P*<0.001; Table 3). Similarly, patients with fatty liver coexisting with MetS had significantly higher risks of elevated ALT, AST and GGT than did patients with fatty liver without MetS (Table 3). The mean ALT, AST and GGT values were higher in patients with fatty liver and T2DM or MetS than in patients with only one disease (Figure 2). Patients with fatty liver coexisting with hypertension and thyroid dysfunction showed similar but not significant results (Table 3). Interestingly, the risk ratios of elevated AST, ALT and GGT did not increase for patients with fatty liver combined with hepatitis B compared with those of patients with fatty liver or hepatitis B alone (Table 3).

**Table 3:** Multivariant analysis of aORs of fatty liver and concomitant diseases status for elevated ALT, AST and GGT levels

	Elevated ALT		Elevated AST		Elevated GGT	
	aOR (95% CI)	*P*-value	aOR (95% CI)	*P*-value	aOR (95% CI)	*P*-value
**Fatty liver and T2DM**						
No fatty liver no T2DM	Reference		Reference		Reference	
Fatty liver (+)	3.13 (3.05–3.21)	<0.001	2.37 (2.28–2.46)	<0.001	2.50 (2.44–2.56)	<0.001
T2DM (+)	1.38 (1.27–1.51)	<0.001	1.30 (1.14–1.48)	<0.001	1.46 (1.37–1.57)	<0.001
Fatty liver (+) T2DM (+)	3.26 (3.10–3.43)	<0.001	3.10 (2.90–3.32)	<0.001	3.74 (3.56–3.92)	<0.001
**Fatty liver and HBV**						
No fatty liver no HBV	Reference		Reference		Reference	
Fatty liver (+)	3.19 (3.11–3.27)	<0.001	2.52 (2.43–2.62)	<0.001	2.57 (2.51–2.62)	<0.001
HBV (+)	2.16 (2.01–2.33)	<0.001	2.75 (2.50–3.02)	<0.001	0.72 (0.66–0.78)	<0.001
Fatty liver (+) HBV (+)	3.46 (3.11–3.85)	<0.001	2.52 (2.18–2.92)	<0.001	1.53 (1.37–1.70)	<0.001
**Fatty liver and MetS**						
No fatty liver no MetS	Reference		Reference		Reference	
Fatty liver (+)	3.08 (2.99–3.16)	<0.001	2.30 (2.20–2.39)	<0.001	2.45 (2.39–2.51)	<0.001
MetS (+)	1.53 (1.45–1.61)	<0.001	1.34 (1.24–1.46)	<0.001	2.35 (2.25–2.46)	<0.001
Fatty liver (+) MetS (+)	3.84 (3.71–3.98)	<0.001	3.05 (2.90–3.20)	<0.001	4.27 (4.13–4.40)	<0.001
**Fatty liver and Hp infection**						
No fatty liver no Hp infection	Reference		Reference		Reference	
Fatty liver (+)	3.18 (3.09–3.27)	<0.001	2.46 (2.36–2.57)	<0.001	2.63 (2.56–2.70)	<0.001
Hp infection (+)	1.05 (1.02–1.08)	0.002	1.06 (1.01–1.11)	0.160	1.08 (1.05–1.11)	<0.001
Fatty liver (+) Hp infection (+)	3.10 (3.00–3.21)	<0.001	2.46 (2.34–2.58)	<0.001	2.63 (2.55–2.72)	<0.001
**Fatty liver and hypertension**						
No fatty liver no hypertension	Reference		Reference		Reference	
Fatty liver (+)	3.19 (3.10–3.27)	<0.001	2.45 (2.35–2.55)	<0.001	2.64 (2.58–2.71)	<0.001
Hypertension (+)	1.22 (1.16–1.28)	<0.001	1.45 (1.36–1.56)	<0.001	1.59 (1.53–1.65)	<0.001
Fatty liver (+) hypertension (+)	3.24 (3.12–3.37)	<0.001	3.12 (2.95–3.29)	<0.001	3.39 (3.27–3.51)	<0.001
**Fatty liver and thyroid dysfunction**						
No fatty liver no thyroid dysfunction	Reference		Reference		Reference	
Fatty liver (+)	3.10 (3.02–3.18)	<0.001	2.41 (2.32–2.50)	<0.001	2.57 (2.51–2.63)	<0.001
Subclinical hyperthyroid (+)	1.09 (1.04–1.14)	<0.001	1.19 (1.11–1.27)	0.466	1.01 (0.98–1.06)	<0.001
Overt hyperthyroid (+)	1.32 (0.93–1.89)	0.122	2.11 (1.43–3.13)	0.670	1.07 (0.79–1.44)	<0.001
Fatty liver (+) Subclinical hyperthyroid (+)	3.42 (3.26–3.59)	<0.001	2.88 (2.70–3.07)	<0.001	2.53 (2.42–2.65)	<0.001
Fatty liver (+) Overt hyperthyroid (+)	3.09 (2.19–4.34)	<0.001	4.20 (2.87–6.17)	<0.001	2.78 (2.04–3.78)	<0.001

Model adjusted for health check year, gender, age, resident, BMI, HBsAg, fatty liver, Hp infection, thyroid function, hypertension, T2DM and MetS.

### Changes in metabolic and lipid profiles with coexisting fatty liver and HBV

Multivariate analysis demonstrated that fatty liver was positively correlated with elevated triglycerides (aOR: 3.39, 95% CI: 3.01–3.81, *P*<0.001), cholesterol (aOR: 1.35, 95% CI: 1.17–1.55, *P*<0.001) and LDL-C (aOR: 1.17, 95% CI: 1.04–1.31, *P*=0.007), whereas HBV was negatively correlated with elevated triglycerides (aOR: 0.46, 95% CI: 0.37–0.59, *P*<0.001), cholesterol (aOR: 0.51, 95% CI: 0.39–0.67, *P*<0.001) and LDL-C (aOR: 0.64, 95% CI: 0.53–0.77, *P*<0.001). Therefore, patients with both fatty liver and HBV exhibited a neutralization of the elevated triglycerides (aOR: 2.14, 95% CI: 1.64–2.8, *P*<0.001), cholesterol (aOR: 0.87, 95% CI: 0.61–1.25, *P*=0.465) and LDL-C (aOR: 1.11, 95% CI: 0.85–1.45, *P*=0.446; Table 4).

**Table 4:** Multivariate analysis of variance for HBV and fatty liver with metabolic and lipid profile

	Model 1		Model 2	
	aOR (95% CI)	*P*-value	aOR (95% CI)	*P*-value
Triglycerides >1.7 mmol/l				
No HBV no fatty liver	Reference		Reference	
Fatty liver (+)	3.51 (3.37–3.66)	<0.001	3.39 (3.01–3.81)	<0.001
HBV (+)	0.59 (0.55–0.64)	<0.001	0.46 (0.37–0.59)	<0.001
HBV (+) Fatty liver (+)	2.01 (1.81–2.24)	<0.001	2.14 (1.64–2.8)	<0.001
HDL-C < 40 mg/dl (♂) or <50 mg/dl (♀)				
No HBV no fatty liver	Reference		Reference	
Fatty liver (+)	2.43 (2.32–2.54)	<0.001	2.47 (2.17–2.8)	<0.001
HBV (+)	0.95 (0.87–1.03)	0.24	0.83 (0.66–1.04)	0.104
HBV (+) Fatty liver (+)	2.54 (2.28–2.84)	<0.001	2.67 (2.03–3.51)	<0.001
Cholesterol > 5.72				
No HBV no fatty liver	Reference		Reference	
Fatty liver (+)	1.54 (1.47–1.62)	<0.001	1.35 (1.17–1.55)	<0.001
HBV (+)	0.66 (0.6–0.72)	<0.001	0.51 (0.39–0.67)	<0.001
HBV (+) Fatty liver (+)	0.97 (0.85–1.12)	0.682	0.87 (0.61–1.25)	0.465
LDL-C > 3.12				
No HBV no fatty liver	Reference		Reference	
Fatty liver (+)	1.27 (1.22–1.32)	<0.001	1.17 (1.04–1.31)	0.007
HBV (+)	0.69 (0.65–0.74)	<0.001	0.64 (0.53–0.77)	<0.001
HBV (+) Fatty liver (+)	1.01 (0.90–1.11)	0.920	1.11 (0.85–1.45)	0.446

Model 1: stratified by sex, urban, gender, year of health check and adjusted for age and BMI.

Model 2: model 1 further adjusted for liver stiffness, albumin and ALT.

## Discussion

In the present study, 357126 patients who underwent physical examinations were automatically selected from the hospital information system of the West China Physical Examination Center. Nearly a quarter of this population were ultrasonographically diagnosed with fatty liver, and most were men living in urban areas. Our results indicated that patients with fatty liver had higher risks of MetS, T2DM and hypertension, and a lower risk of HBV. Fatty liver coexist.ing with T2DM, MetS and thyroid dysfunction conferred a significantly higher risk of elevated transaminase levels. Fatty liver was positively correlated with triglycerides, cholesterol and LDL-C, whereas coexisting HBV had a neutralizing effect on lipid metabolism.

Regarding fatty liver and HBV, a meta-analysis that included 30 studies suggested that HBV infection was inversely associated with MetS prevalence, and among MetS components, elevated triglycerides had the strongest inverse relationship with HBsAg positivity [35], which was similar to our results shown in Table 4. A recent review also revealed an inverse relationship between HBV and increased triglycerides [36]. The liver is the main organ for lipid metabolism, and factors that can affect liver function, especially HBV infection, may be involved in liver lipid synthesis and metabolism [37]. Both previous studies and our research suggest that HBV may prevent, rather than promote, lipid deposition in hepatocytes. Regarding the mechanism, previous studies demonstrated that HBV X protein (HBx) induces the transcriptional activation of peroxisome proliferator-activated receptor γ (PPARγ) [38], and the activation of PPARγ gene expression boosts an increase in circulating adiponectin levels [39,40]. Adiponectin protects against IR; thus, it is inversely associated with BMI, T2DM, and several metabolic disorders, such as cardiovascular disease and atherosclerosis [41]. Additionally, a study reported that HBV may influence lipid deposition and lipid droplet size in hepatocytes by decreasing the expression of cell death-inducing DFF45-like effectors (CIDEs) B and C (CIDEB and CIDEC), which are involved in lipid droplet expansion for improving lipid storage [42]. Thus, the increased risk and progression of both fatty liver and coronary disease should be carefully monitored when treating patients with chronic HBV and NAFLD. Apart from reports that HBV decreased lipogenesis, several other studies showed that HBV infection also increased lipid biosynthesis [43–45]. In response to this, some researchers have proposed that HBx may interact with the liver X receptor α (LXRα) and enhances the binding of LXRα to the LXR-response element, thus up-regulating sterol-regulatory element-binding protein 1 and fatty acid synthase [46,47]. Overall, the changes in lipid metabolism involved in NAFLD among populations with CHB require further investigation. In addition to lipid metabolism changes, we found that patients with coexisting fatty liver and HBV infection did not have an increased risk of liver dysfunction compared with patients who had either disease alone. Despite a lot of studies have described the complex pathophysiological states, the complex mechanistic interactions between NAFLD and HBV infection remains uncertain [26]. Therefore, the effects of NAFLD on CHB virology and histology and *vice versa* require further study.

Considering fatty liver and thyroid dysfunction, increasing data show a higher prevalence of thyroid dysfunction in the form of overt or subclinical hypothyroidism among patients with NAFLD/NASH [20]. Our results also suggest that patients with hypothyroidism are at higher risk for associated fatty liver disease and are more prone to liver function abnormalities. However, multiple studies have yielded conflicting data regarding whether hypothyroidism can predict the severity of fatty liver. Two studies confirmed an association between the severity of NAFLD and hypothyroidism [48,49], while others found no statistically significant association between hypothyroidism, simple steatosis and NASH [50–52]. These studies were retrospective cohort studies. Sex and age of the included population did not significantly differ, but different studies drew different conclusions, possibly owing to the number of populations, the study location and screening criteria, and the analysis methods. In summary, the current findings regarding whether NAFLD/NASH is associated with thyroid dysfunction remain controversial and it should be further verified in placebo-controlled clinical trials.

Finally, regarding fatty liver and Hp infection, some studies have suggested that Hp infection may be a risk factor for fatty liver disease, because Hp infection may directly or indirectly induce IR in NAFLD [12,53,54], and IR is an independent risk factor of NAFLD [55]. Several studies demonstrated that chronic inflammation due to Hp infection can increase the levels of inflammatory cytokines such as tumor necrosis factor (TNF)-α and interleukin (IL)-6, which can activate a series of kinases such as IKK/NF-κB and JNK, eventually trigger IR by up-regulating Ser-phosphorylation [56–59]. In addition, Hp infection can also inhibit the release of leptin from white adipose tissue [60], which can decrease *de novo* lipogenesis in patients with lipodystrophy and eventually reduce very low-density lipoproteins (VLDL) production [61–63]. Furthermore, studies have suggested that Hp invasion of the intestinal mucosa can increase intestinal permeability and intestinal flora disorders, and promote bacterial endotoxins (especially lipopolysaccharide) through the portal vein into the liver and promote inflammatory responses, resulting in decreased lipoprotein activity, followed by dyslipidemia [64,65]. Despite the positive association between Hp and NAFLD reported in some studies, other studies deny their relationship. For example, several clinical studies from Asia (including Japan, Korea and China) have concluded that Hp infection is unassociated with NAFLD [66–70]. After comparing the differences in these studies, we found that the factors of race, region and number of included populations can affect the results of the study. In our study, we found that Hp infection was not significantly associated with fatty liver; thus, more clinical evidence and basic research are needed.

The present study had some limitations. First, the population of patients who underwent a physical examination at West China Hospital, which was not a random sampling and thus may have biased the results. Therefore, the HBV prevalence was higher in the present study, and some patients with other liver diseases, such as autoimmune liver diseases or hepatitis C virus infection, were not screened out. Second, no secondary tests will be performed in a large population-based study; thus, it will be difficult to validate our data with long-term follow-up. Third, this research was cross-sectional; thus, the data only show an association between fatty liver and coexisting diseases and between coexisting diseases and abnormal liver transaminase levels but not a causal link. The underlying mechanism for this remains largely unknown. Further prospective and basic studies are required to verify this molecular mechanism. Finally, previous antiviral treatments and HBV DNA were putative confounding factors for elastography measurements and lipid profiles. Unfortunately, we lacked these data from the health examination program.

In conclusion, patients with fatty liver are more likely to have coexisting MetS, T2DM and hypertension, but fatty liver is less common in patients with hepatitis B. Additionally, because fatty liver coexisting with T2DM, MetS and thyroid dysfunction conferred a significantly higher risk for elevated transaminase, more attention should be paid in clinical practice to protecting liver function in these patients. Finally, when fatty liver is combined with HBV, it may exert a neutralizing effect on lipid metabolism. Thus, lipid alterations should be monitored in patients with both fatty liver and HBV during antiviral treatment for HBV.

## Data Availability

The datasets used and analyzed in the present study are available upon reasonable request.

## Abbreviations

ALT: alanine transaminase
aOR: adjusted OR
AST: aspartic transaminase
BMI: body mass index
CHB: chronic hepatitis B
CI: confidence interval
GGT: γ-glutamyl transferase
HBsAg: hepatitis B s antigen
HBx: hepatitis B virus X protein
HBV: hepatitis B virus
Hp: *Helicobacter pylori*
IR: insulin resistance
JNK: c-Jun N-terminal kinase
LDL: low-density lipoprotein
LDL-C: low-density lipoprotein cholesterol
MetS: metabolic syndrome
NAFLD: nonalcoholic fatty liver disease
NASH: non-alcoholic steatohepatitis
NF-κB: nuclear factor kappa-B
OR: odds ratio
PPARγ: peroxisome proliferator-activated receptor γ
TSH: thyroid stimulating hormone
T2DM: type 2 diabetes mellitus
